# Exploring the metabolic and physiological roles of *HQT* in *S. lycopersicum* by gene editing

**DOI:** 10.3389/fpls.2023.1124959

**Published:** 2023-03-31

**Authors:** Fabio D’Orso, Lionel Hill, Ingo Appelhagen, Tom Lawrenson, Marco Possenti, Jie Li, Wendy Harwood, Giorgio Morelli, Cathie Martin

**Affiliations:** ^1^ Council for Agricultural Research and Economics (CREA), Research Centre for Genomics and Bioinformatics, Rome, Italy; ^2^ John Innes Centre, Norwich Research Park, Norwich, United Kingdom

**Keywords:** HQT, caffeoylquinic acids, genome editing, *S. lycopersicum*, metabolic engineering, phenylpropanoid pathway, abiotic stresses, UV-light stress

## Abstract

The most abundant phenolic compound in Solanaceous plants is chlorogenic acid (CGA), which possesses protective properties such as antimicrobial and antioxidant activities. These properties are particularly relevant when plants are under adverse conditions, such as pathogen attack, excess light, or extreme temperatures that cause oxidative stress. Additionally, CGA has been shown to absorb UV-B light. In tomato and potato, CGA is mainly produced through the HQT pathway mediated by the enzyme hydroxycinnamoyl-CoA:quinate hydroxycinnamoyl transferase. However, the absence of natural or induced mutants of this gene has made it unclear whether other pathways contribute to CGA production and accumulation. To address this question, we used CRISPR technology to generate multiple knock-out mutant lines in the tomato HQT gene. The resulting *slhqt* plants did not accumulate CGA or other caffeoylquinic acids (CQAs) in various parts of the plant, indicating that CQA biosynthesis depends almost entirely on the HQT pathway in tomato and, likely, other Solanaceous crops. We also found that the lack of CGA in *slhqt* plants led to higher levels of hydroxycinnamoyl-glucose and flavonoids compared to wild-type plants. Gene expression analysis revealed that this metabolic reorganization was partly due to flux redirection, but also involved modulation of important transcription factor genes that regulate secondary metabolism and sense environmental conditions. Finally, we investigated the physiological role of CGA in tomato and found that it accumulates in the upper epidermis where it acts as a protector against UV-B irradiation.

## 1 Introduction

Caffeoylquinic acids (CQAs) are important naturally occurring hydroxycinnamic acid conjugates present in many higher plants. In particular, chlorogenic acid (CGA, 5-CQA) is the most abundant phenolic compound in Solanaceous species such as potato, tomato, aubergine, tobacco, as well as important crops such as plum, apple and coffee. CGA has been reputed to have many important functions in plants protecting against biotic and abiotic stresses. Tomato leaves with enhanced CGA levels showed lower sensitivity to *Pseudomonas syringae* infection (Niggeweg et al., 2004) and CGA is associated with the tolerance of tomato fruit to *Alternaria alternata* by inhibiting alternariol biosynthesis (Wojciechowska et al., 2014). Moreover Clé et al. (2008) showed that CGA plays a key role in plant protection against harmful UV-B light. CGA is a powerful antioxidant phenolic compound that helps neutralise the accumulation of reactive oxygen species (ROS). This limits cell damage and protects against oxidative stress caused by harsh environmental conditions, such as high light, extreme temperatures, drought, salt and heavy metals (Soviguidi et al., 2022).

CGA is also important because of its reputed beneficial properties for human health, including antioxidant, anticarcinogenic, anti-inflammatory, antibacterial, antidiabetic properties (Tajik et al., 2017).

**Figure 1:**
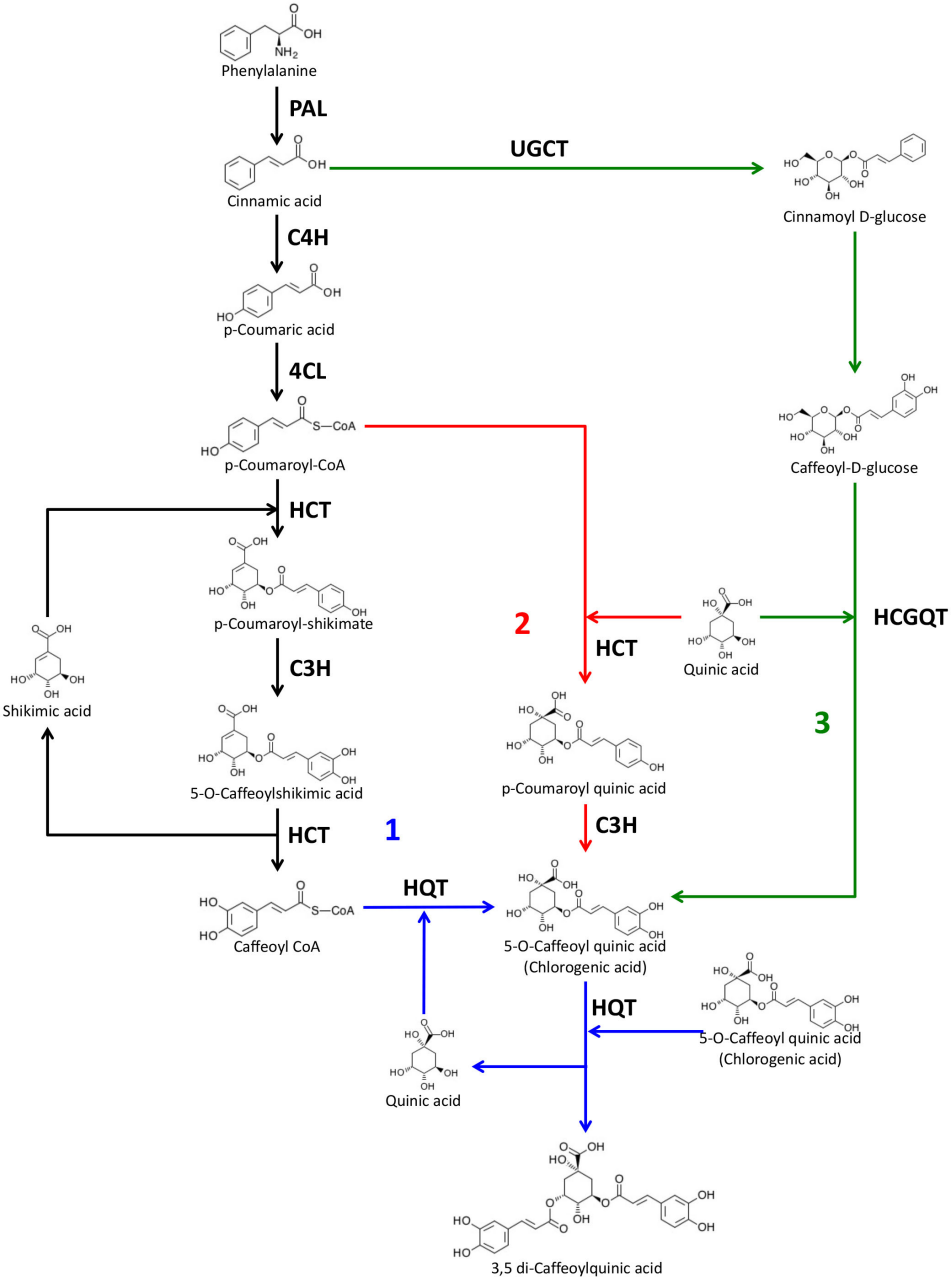
Proposed biosynthetic pathways for chlorogenic acid and dicaffeoylquinic acids. The three possible pathways for CGA biosynthesis proposed in plants are indicated as 1, 2 and 3. PAL, phenylalanine ammonia lyase; C4H, cinnamate 4-hydroxylase; 4CL, 4-hydroxycinnamoyl-CoA ligase; HCT, hydroxycinnamoyl-CoA shikimate/quinate hydroxycinnamoyl transferase; HQT, hydroxycinnamoyl-CoA quinate hydroxycinnamoyl transferase; C3H, *p*-coumaroyl ester 3′-hydroxylase; UGCT, UDP glucose:cinnamate glucosyl transferase; HCGQT, hydroxycinnamoyl D-glucose:quinate hydroxycinnamoyl transferase. Source of molecule images: KEGG database (Kanehisa et al., 2023).

For these reasons, understanding the CQA biosynthetic pathway is important, especially in Solanaceous species like tomato, potato, pepper and aubergine which represent horticultural crops of worldwide importance. Three distinct pathways have been proposed for CGA production in plants (**Figure 1**). Route 1 involves the enzyme hydroxycinnamoyl-CoA:quinate hydroxycinnamoyl transferase (HQT) which converts caffeoyl-CoA and quinic acid to CGA (Stöckigt and Zenk, 1974; Rhodes and Wooltorton, 1976; Niggeweg et al., 2004). The second route depends on the synthesis of *p-*coumaroylquinate by an acyl transferase (HCT), followed by hydroxylation by *p*-coumaroyl-3’-hydroxylase C’3H (Ulbrich and Zenk, 1979; Hoffmann et al., 2003; Mahesh et al., 2007). Finally, a third route has been proposed in sweet potato (*Ipomea batatas*) where caffeoyl glucoside serves as an activated intermediate (Villegas and Kojima, 1986).

The HQT-mediated pathway is considered the most important one for CGA production in many plants and its role has been established in several species, including tobacco, tomato, potato and artichoke (Niggeweg et al., 2004; Payyavula et al., 2015; Sonnante et al., 2010). In tomato the silencing of SlHQT resulted in a significant reduction of CGA and the overexpression of SlHQT led to an accumulation of CGA (Niggeweg et al., 2004). SlHQT is also involved in di-caffeoylquinic acid biosynthesis by its secondary chlorogenate:chlorogenate transferase activity in the vacuole (Moglia et al., 2014).

Unfortunately, despite many advances in the understanding of the role of HQT in CQA biosynthesis, the relative importance of the HQT-mediated pathway remains to be demonstrated. Indeed, because of the lack of natural or induced mutations of this gene, no one has yet evaluated the effect of the complete absence of HQT on caffeoylquinate metabolism nor on the physiological roles of CGA in plants which accumulate high levels of this metabolite.

Among several techniques available for generating targeted knock-out mutations, the *clustered regularly interspaced short palindromic repeats* (CRISPR)/Cas9 system is particularly effective and has been widely used due to its efficacy and ease of use in the experimental design and the building of vectors for editing (Puchta, 2017).

In this work we generated knock-out mutations in the SlHQT gene and characterized CGA accumulation in different tomato organs. We assessed the effect of *HQT* ko mutations on phenylpropanoid metabolism, on the expression of genes encoding enzymes in the phenylpropanoid pathway and on regulatory genes under normal and abiotic stress conditions. In addition, the *hqt* mutants enabled us to investigate the histolocalization of CQAs in tomato leaves and to understand their roles in photoprotection against UV light.

## 2 Materials and methods

### 2.1 CRISPR construct and tomato transformation

To target the *HQT* gene (Solyc07g005760) we designed two single guide RNAs (sgRNAs), one targeting the 5’UTR: CCTTATCTCTTTAGCTCTTTCTC and the other one targeting the coding sequence: GAAGTTAATTGTAATGGTGAAGG (PAM underlined).

These sequences were fused to the chimeric single-guide RNA (Jinek et al., 2012) by PCR. The primers sgHQT_A_FW and sgHQT_B_FW coupled with the sgRNA_REV, were used to amplify the *HQT* sgRNAs using the plasmid pICSL70001 as template. Primer sequences are listed in the **Table S1**. The PCR products A and B were gel purified using a Qiagen gel extraction kit and they were cloned in Level 1 cloning vectors pICH47732 (position 1) and pICH47742 (position 2), respectively, together with U6-III promoter (pICSL90001) by BsaI-mediated DIG-LIG reaction (GoldenGate cloning system). sgRNA expression cassettes in level 1 vectors were assembled together with the end linker (pICH41744) in the Level 2 binary vector pICSL002203 already containing the expression cassettes Pnos-NPTII-ocsT and 2x35S-cas9-nosT by BbsI-mediated DIG-LIG reaction. The final construct was introduced into *Agrobacterium tumefaciens* AGL-1. Tomato (*Solanum lycopersicum*) plant transformation and regeneration was carried out using the cultivar Money Maker by standard regeneration methods (Fillatti et al., 1987).

### 2.2 Genotyping

Genomic DNA was extracted from leaves of T0 and T1 plants using DNeasy Plant Mini Kits (Qiagen).

*HQT* and *HCT* genomic regions were amplified using around 10 ng of genomic DNA by Phusion High-Fidelity DNA Polymerase (Thermo Scientific). Amplicons were gel purified by QIAquick Gel Extraction Kit (Qiagen) and analysed by Sanger sequencing. Plants where the T-DNA had segregated out were selected in the T1 generation. The presence of the T-DNA was monitored by amplification of cas9 directly from leaf tissue using Phire Plant Direct PCR Master Mix following the Dilution & Storage protocol (Thermo Fisher Scientific). All primers for *HQT*, *HCT* and T-DNA genotyping are listed in the **Table S2**.

### 2.3 Expression analysis

Total RNA was extracted from tomato leaflets using RNeasy Plant Mini kit (Qiagen) according to the manufacturer’s instructions. RNA concentration and quality were verified by spectrophotometric measurements using NanoDrop ND-1000. 1 μg of total RNA was treated with DNase for a complete removal of genomic DNA contamination (DNase I Amplification Grade SIGMA-Aldrich -Catalog Number AMPD1). First-strand cDNA synthesis was performed using High Capacity cDNA Reverse Transcription Kits (Applied Biosystems).

qPCR experiments were performed on a LightCycler^®^ 480 II detection system using LightCycler^®^ 480 SYBR Green I Master (Roche) with the following cycle program 5 min at 95°C, followed by 45 cycles of 15 sec at 95°C, 15 sec at 60°C and 1 min at 72°C. Gene-specific primers are listed in the **Table S3**. Tomato elongation factor 1-alpha (SlEF1A) gene was used as reference gene to calculate relative expression (Livak and Schmittgen, 2001).

### 2.4 HQT activity assay

Tomato leaves from WT and *hqt* plants homozygous for the four *hqt* alleles were powdered with a pestle and mortar in liquid nitrogen, the powder was immediately extracted with 0.1 M sodium phosphate buffer, pH 7.4 containing 10 mM DTT and 10% of PVPP (Sigma). The mixture was incubated at 4°C for 40 minutes in agitation and then centrifuged at 4°C at 13,000 rpm. 1 ml of supernatant was transferred into Centricon-10 filter device (Amicon) and filtrated by centrifugation, the retentate was washed twice with 1 ml of 0.1 M sodium phosphate buffer, pH 7.4 containing 10 mM DTT. The final retentate constituted the crude extract used for HQT activity measurements.

HQT enzyme activity was assayed in the reverse direction spectrophotometrically at 30°C. The reaction mixture contained in 1.5 ml: 100 mM phosphate buffer pH 7.0, 1mM EDTA, 400 μM CGA and 30 μl of crude extract. The reaction was started by addition of 400 μM CoA (or water as a negative control) and the synthesis of Caffeoyl-CoA was monitored by measuring the increase in absorbance at 360 nm (Rhodes and Wooltorton, 1976). Protein content was quantified by the Bradford method (Bradford, 1976) using bovine serum albumin as an internal standard.

### 2.5 Total anthocyanin quantification

For sample preparation for metabolic analyses, 10 mg of powdered and freeze-dried plant material was extracted with 500 μl of 80% MetOH at 4°C in agitation, the samples were centrifugated at the max speed for 10 minutes at 4°C. The supernatant was collected and transferred in a new tube. The pellet was used to repeat the extraction with 500 μl of 80% MetOH incubated at 4°C for 3 hours. After centrifugation (max speed for 10 minutes), the supernatant was collected and mixed with the previous one.

For anthocyanin quantification 375 μl of extract were acidified with 125 μl of 2.4% HCl solution and mixed with 500 μl of chloroform, the sample was vortexed and the centrifuged at the max speed for 5 minutes. Total anthocyanins in the aqueous phase were determined spectrophotometrically and expressed as mg of petunidin-3-(*p*-coumaroyl rutinoside)-5-glucoside per g dry weight (Butelli et al., 2008).

### 2.6 CQA analysis

For CQAs and diCQAs the samples were run on a Nexera/Prominence UHPLC system attached to a 2020 single quadrupole mass spectrometer (both from Shimadzu). Separation was on a 100×2.1mm 2.6μ EVO C18 column (Phenomenex) using the following gradient of acetonitrile (B) versus 0.1% formic acid in water (A), run at 0.55 ml.min^-1^ and 40°C: 5% B over 0.0-11.0 min, 25% B over 11.0-11.5 min, 80% B over 11.5-14.0 min, 5% B over 14.0-18.00 min. Samples were kept at 15°C in the autosampler prior to injection. The PDA collected spectra from 200-800 nm at 6.25Hz with a time-constant of 0.24 sec. The MS was equipped with a dual ion source, used in electrospray mode (no APCI), with spray chamber conditions of 250°C desorbation line temperature, 200°C heat block, 1.5l.min^-1^ nebulizer gas, and 15l.min^-1^ drying gas.

CQAs and diCQAs were analysed by single ion monitoring event in negative mode monitoring the following ions for 0.25 sec in total: m/z 353 for CQA and isomers and m/z 515 for diCQAs.

### 2.7 Untargeted metabolomic analysis of leaves

The extracts were analysed chromatographically on a Shimadzu Nexera/Prominence UHPLC system attached to an ion-trap ToF (IT-ToF) mass spectrometer. Separation was on a 100×2.1mm 2.6μ Kinetex EVO C18 column (Phenomenex) using the following gradient of acetonitrile (B) versus 0.1% formic acid in water (A), run at 0.5ml.min^-1^ and 40°C: 2% B over 0.0-4.5 min, 10% B over 4.5-8.0 min, 30% B over 8.0-11.0 min, 90% B over 11.0-11.40 min and 90-2% over 11.40-15.00 min. Injection volume was 10 μl.

The photo diode array detector (PDA) was set to collect spectra from 200-600 nm at 6.25 Hz with a time constant of 0.08 sec. The MS was set up to collect either positive or negative spectra from *m/z* 200-2000 with a maximum ion accumulation time of 20 msec and automatic sensitivity control set to a target of 70% optimum base peak intensity. This allowed the collection of one spectrum every 0.13 sec. Spray chamber conditions were 1.5l.min^-1^ nebulizer gas, 250°C curved desorbation tube temperature, 300°C heat block, and drying gas “on”. The instrument was calibrated using sodium trifluoroacetate cluster ions according to the manufacturer’s instructions, immediately before use. For MS2 spectra, the ion accumulation time was fixed at 20 msec; the instrument was set to collect two spectra for each mass, before ignoring that mass in favour of the next most abundant ion for 3 sec. The precursor ion isolation width was *m/z* 3.0, and fragmentation was at 50% collision energy and 50% collision gas.

Untargeted screening for ‘peaks changed’ was caried out by Shimadzu’s ProfilingSolution software. The software was set to look for all peaks with a height of more than 150,000 counts and to regard peaks from different samples as the same chemical if their mass differed by not more than 20 mDa, and their retention time by not more than 0.1 min. The isomer valley setting was 20%.

Peak changes between *hqt* mutants and WT were assessed first by statistical analysis using ProfilingSolution software and then by Benjamini-Hochberg test for false discovery rate. For the peaks that passed the Benjamini-Hochberg false discovery rate at 5%, we attempted the identification of the corresponding compounds.

### 2.8 Targeted metabolite profiling

For LC-UV/MS analysis of targeted metabolites, extracts were run on the Shimadzu Nexera/Prominence UHPLC attached to an IT-ToF mass spectrometer. Separation was on a 100×2.1mm 2.6μ Kinetex EVO C18 column (Phenomenex) using the following gradient of acetonitrile (B) versus 0.1% formic acid in water (A), run at 0.5ml.min^-1^ and 40°C: 2% B over 0.0-3.0 min, 10% B over 3.0-13.0 min, 30% B over 13.0-18.0 min, 90% B over 18.0-19.00 min and 90-2% over 19.00-23.10 min.

Detection was by UV/visible absorbance and positive mode electrospray MS. The diode array detector collected spectra from 200-600nm at 12.5Hz with a time constant of 0.08sec. The MS collected spectra from *m/z* 200-2000 with automatic sensitivity control set to a target of 70% optimal base peak intensity. The instrument also collected data-dependent MS2 spectra of the most abundant precursor ions from *m/z* 50-2000 at 50% collision energy, 50% collision gas, and an isolation width of *m/z* 3.0, and a fixed ion accumulation time of 10msec. After a precursor ion had been selected, it was ignored for 1 sec in favour of the next most abundant precursor ion. Spray chamber conditions were 250°C curved desorbation line, 300°C heat block, 1.5 l.min^-1^ nebulizer gas, and drying gas “on”. The instrument was calibrated using sodium trifluoroacetate cluster ions according to the manufacturer’s instructions, immediately before use. Retention times and mass spectral data for the compounds identified are shown in **Tables S4**, **S5**. Compound identification is based on UV-spectral and MS fragmentation features and thus should be considered as tentative identification.

### 2.9 Plant material and growth conditions

*Solanum lycopersicum* cv. Moneymaker was used in this work. Plants were grown in controlled conditions between 25-28°C and natural light (unless differently indicated) in the glasshouse at John Innes Centre (Norwich, UK). For high light treatment, plants were grown under natural light supplemented with artificial light by Heliospectra Elixia LED lamps (600W) turned on for 16h per day.

For cold stress experiments, WT and *hqt* mutants were germinated on water-imbibed filter paper, after 7-10 days the seedlings were transferred to soil in 10 cm diameter pots and placed in growth chambers with a long-day photoperiod (16 h light, at 100 μmol m^-2^ s^-1^ photon flux density) at 28°C for 3 weeks. After that, the plants were treated with cold temperature (15°C) for 11 days. Terminal leaflets of well expanded leaves were collected before cold stress (as the control condition) and after 11 days for metabolite and gene expression analysis.

### 2.10 Confocal laser scanning microscopy for histolocalization of CQAs in tomato leaf

Images were taken with a Leica SP5II confocal microscopes equipped with HCX PL APO CS 63.0x1.20 water immersion objective (Leica Microsystems GmbH). Fluorescence was detected by hybrid detector Hyd3. Emission spectra were recorded with 3 nm step size and 10 nm bandwidth after excitation at 405 nm laser. CQA/diCQA fluorescence was subsequently recorded in the range of 440 nm – 560 nm after excitation at 405 nm, to avoid detection of chlorophyll auto-fluorescence. The LAS AF software (version 2.7.3) was used for image acquisition and intensity measurements.

### 2.11 UV-light stress

We used the primary leaflets (length ~8 cm and width ~4 cm) of well-expanded leaves. Ten leaflets per genotype were collected from plants grown in the greenhouse, sterilized with 10% of commercial bleach for 10 minutes in plastic sterile boxes in gentle agitation, after they were washed several times with sterile water and then placed in petri dishes (one leaflet per dish) on MS medium with 3% sucrose. The plates were left in the growth chamber over-night, and the day after five plates per genotype were placed without the lid 20 cm below an inverted short wavelength transilluminator and the leaflets were exposed to UV-B light (3.2 mW/cm^2^) for 30 minutes. The plates were then closed and the leaflets were pictured 4 days after the exposure to UV light. In parallel, the same number of leaflets were kept in the growth chamber as the mock experiment.

## 3 Results

### 3.1 Screening and selection of *slhqt* mutant plants

**Figure 2:**
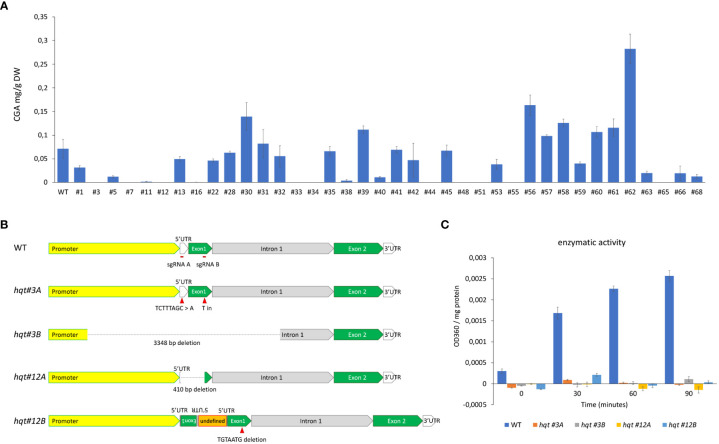
Characterization of *HQT*-edited plants. **(A)** CGA levels in tomato fruit (Br+10: 10 days past breaker) of T0 plants transformed with CRISPR construct. The data represent the means ± SE (n = 3) **(B)** Genotypic characterization of selected lines. **(C)** hydroxycinnamoyl-CoA quinate hydroxycinnamoyl transferase activity evaluation in crude extracts of WT and *hqt* plants, activity calculated as difference in OD360 between CoA and mock reactions normalized for protein concentration. The data represent the means ± SE (n = 3).

With the intention of targeting the gene encoding HQT to better understand its contribution to CQA biosynthesis in tomato, we designed a CRISPR/Cas9 construct with two sgRNAs, one in the 5’UTR and the other in the first exon (see Material and Methods). Plants regenerated after agrobacterium-mediated transformation were analysed by HPLC/MS to quantify the level of CGA (**Figure 2A**). Based on the results of metabolite analysis, we picked two lines (#3 and #12) with no detectable CGA in their fruit (Br+10 stage). Genotypic analysis by PCR and sequencing in T0 and T1 plants revealed that both lines carried biallelic knock-out mutations.

The allele *hqt #3A* brought two different mutations on the two target sites: (i) a substitution of TCTTTAGC sequence with an A in the 5’UTR, (ii) an insertion of a T between the 282^nd^ and the 283^rd^ nt of the coding sequence (**Figure 2B**), resulting in a frame shift of the coding sequence and a truncated peptide. The allele *hqt #3B* showed a 3348 bp deletion: this region includes 1610 bp of the promoter, all 5’UTR (137 bp), all the first exon (424 bp) and 1177 bp of the first intron. The line *hqt #12* had one allele (*hqt #12A*) with a 410 nt deletion between the two guides, while the second allele (*hqt #12B*) had a complex rearrangement (**Figure 2B**). In particular, in *hqt #12B*, a TGTAATG sequence was deleted from the target of guide 1B on the exon 1; on the target of guide 1A a genomic portion of *HQT* gene, which includes part of the 5’UTR and part of the exon 1, had been inserted generating an inverted repeat; also, between the two inverted repeats there was an undefined sequence of about 500 bp.

To generate T-DNA free plants we screened T1 plants for the presence of the cas9 gene by PCR. We found 5% for line #3 and 25% for line #12 were without cas9. These segregation ratios were compatible with double and single T-DNA insertions in the T0 line *hqt#3* and *hqt#12*, respectively. All experiments were carried out by using homozygous plants for *hqt#3A* and *hqt#12A* alleles (unless differently indicated in the figure’s legends), hereafter simply indicated as *hqt#3* and *hqt#12*.

No off-target mutations were found in the coding sequence of *HCT* gene in these *hqt* mutant lines.

The complete inactivation of HQT enzyme in the selected *hqt* lines was then evaluated by hydroxycinnamoyl-CoA quinate hydroxycinnamoyl transferase activity assays in crude extracts from leaves of WT and *hqt* plants. No activity was detected in these mutant plants (**Figure 2C**).

### 3.2 CQA content in WT and *hqt* tomato plants

**Figure 3:**
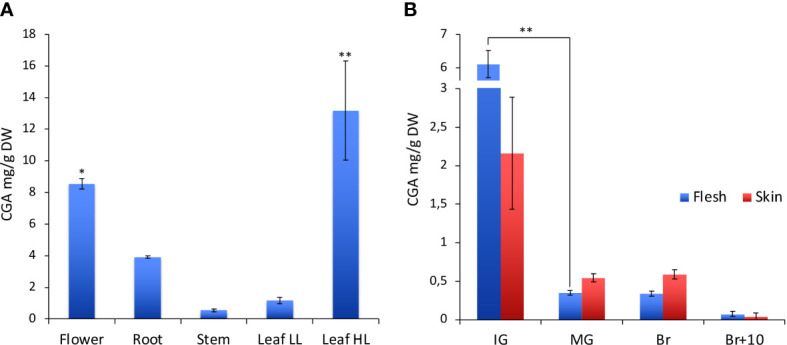
Characterization of CGA in WT tomato plants. **(A)** CGA content in different organs of the tomato plant. In leaves CGA was quantified under two different light conditions: low light (LL) and high light (HL). **(B)** CGA content at different fruit development and ripening stages (IG, immature green; MG, mature green; Br, breaker; Br+10, 10 days past breaker). For CGA content among tomato organs statistical significance was assessed by one-way ANOVA analysis followed by Dunnet’s tests comparing all samples vs leaf LL, *P < 0.05, **P < 0.01. For CGA content in tomato fruit at several stages of development and ripening multiple comparisons among stages (*P < 0.05, **P < 0.01) and tissues have been performed by one-way ANOVA analysis followed by Bonferroni’s test.

To evaluate the role of HQT in the biosynthesis of CGA, we analysed its distribution in different organs (leaf, stem, root, flower and fruit at different stages of development and ripening) in WT and mutant plants (**Figure 3** and **Table 1**). Consistent with its role in protection of photosynthetic apparatus, we found that CGA accumulated in stems and leaves of WT plants; this analysis also showed a conspicuous amount of CGA in WT flowers and roots (**Figure 3A**).

**Table 1:** Chlorogenic acid quantification in WT and *hqt* tomato plants.

CGA quantification (mg/g DW)							
						Fruit (Br)	
	Flower	Root	Stem	Leaf LL	Leaf HL	Flesh	Skin
WT	8.56 ± 0.33	3.91 ± 0.08	0.54 ± 0.11	1.17 ± 0.20	13.19 ± 3.14	0.34 ± 0.03	0.59 ± 0.06
*hqt#3*	trace*	trace*	0	0	0.151 ± 0.06 (1.1%)^§^	0	0
*hqt#12*	trace*	trace*	0	0	0.119 ± 0.01 (0.9%)^§^	0	0

Flower, root, stem and leaf LL samples are from plants grown under normal light condition. Leaf HL samples are from plants grown with supplemental artificial light.

*unquantifiable amount detected by MS.

^§^ratio between hqt and WT plants.

Mean ± Standard Error, n=3.

LL, Low Light.

HL, High Light.

Br, Breaker.

We also tested whether the CGA content in the leaves could vary according to environmental light conditions. In particular, plants grown in the greenhouse under natural light (low light - LL) accumulated about 1.2 mg/g DW of CGA in the leaves, whereas plants grown under supplemental artificial light (high light - HL) increased the CGA content to about 13 mg/g DW (**Figure 3A**).

Fruits were not particularly rich in CGA, although production was regulated during development and ripening (**Figure 3B**); the maximum content of chlorogenic acid was reached at the immature green stage (IG), it decreased during fruit development and during ripening (statistically significant differences only for the comparison IG vs MG). At the IG stage the CGA amount was higher in the flesh than in the skin (even if not statistically significant), whereas at other stages the contents of CGA in flesh and skin were quite similar.

To investigate the role of HQT in CQA production, we examined whether *hqt* knock-out mutations led to a complete absence of CGA and other CQAs in various parts of the plant. We analysed fruit at breaker stage (Br) and neither *hqt* lines (#3 and #12) showed any trace of CGA (**Tables 1**, **S6**) or other CQAs (**Table S7**), confirming our observations in T0 fruit (Br+10 stage). The same was true for stems and leaves in LL conditions. In contrast, in other organs like flowers and roots (which are richer in CGA in WT plants) we found traces of CGA, the amounts of which were so low as to be unquantifiable (**Table 1**). To assess the residual percentage of CGA synthetized in *hqt* plants compared to WT plants, we measured this compound in tomato leaves from plants grown under HL conditions which induced the phenylpropanoid pathway. In these conditions *hqt* mutants showed very small amounts of CGA, sufficient to attempt quantification. When compared with WT, this residual CGA production in *hqt#3* and *hqt#12* lines amounted to 1.1% and 0.9%, measured by PDA (**Table 1**), and 0.2 and 0.18%, measured by MS (**Table S6**), respectively.

Taken together, our results show that HQT is the enzyme responsible for almost all CGA biosynthesis in tomato. However, the trace of CGA detected in some samples suggested that a second enzymatic activity might be able to produce tiny amounts under some conditions. This minor activity is most likely attributable to HCT (Hoffmann et al., 2003) which is consistent with its transcript levels in flowers and roots (Sato et al., 2012).

### 3.3 Effect of CGA depletion on leaf metabolism

Chlorogenic acid is the most abundant phenolic compound in Solanaceous species like tomato, potato and aubergine (Whitaker and Stommel, 2003; Niggeweg et al., 2004; Payyavula et al., 2015). It has been shown that modulation of HQT expression (and consequently CGA levels in leaves) alters the total amount of phenolics, with clear enrichment or depletion of specific compounds (Clé et al., 2008) even in an organ specific way (Payyavula et al., 2015). Therefore, the alteration of HQT activity is likely to have significant effects on phenylpropanoid metabolism.

**Figure 4:**
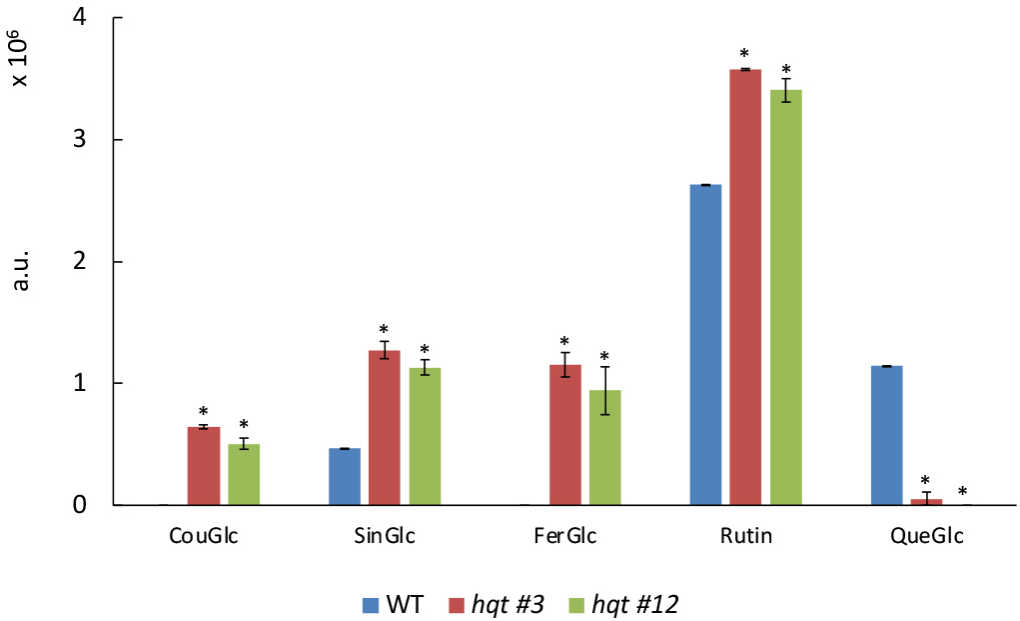
Targeted phenylpropanoid profiling of WT and *hqt* tomato plants grown in greenhouse. Levels of coumaroyl-glucose, sinapoyl-glucose, feruloyl-glucose, rutin and quercetin-glucose in leaves of tomato plants grown in greenhouse under natural light. The data show means ± SE (n = 3). Statistical significance was assessed by one-way ANOVA analysis followed by Dunnet’s tests for *hqt* lines vs WT, *P < 0.01. CouGlc, coumaroyl-glucose; SinGlc, sinapoyl-glucose; FerGlc, feruloyl-glucose; Rut, rutin; QueGlc, quercetin-glucose.

To characterize the effect of the absence of HQT activity better, we analysed the soluble phenolic compounds extracted from WT and *hqt* tomato leaves by LC-MS/MS. To this end, we carried out an untargeted analysis finding the peaks of ions generated after positive or negative ionization, focusing on peaks with significant differences between WT and both *hqt* mutant plants (**Figure 4** and **Tables S4**, **S8**). The most significant difference in soluble phenolics was the higher accumulation of some hydroxycinnamoyl-glucoses, such as sinapoyl-glucose, coumaroyl-glucose and feruloyl-glucose in *hqt* plants. This suggested that the block in CQA biosynthesis caused the accumulation of hydroxycinnamoyl-CoA intermediates and their subsequent transesterification with glucose. Among flavonols, we found that the rutin concentration increased and quercetin-glucose (which could be a fragmentation product of a more complex unidentified minor flavonol) decreased.

These results suggested that the block in CGA production resulted in a significant reorganization of the equilibria between phenylpropanoids by redirecting metabolic flux towards the synthesis of other compounds which use caffeoyl-CoA, the precursor of CGA. Interestingly, from our analysis we did not observe any alteration of quinate derivatives in *hqt* lines, suggesting that apparently the quinate precursor is not redeployed into other metabolic branches.

### 3.4 Effects of CGA depletion on leaf metabolism at normal and low temperatures

**Figure 5:**
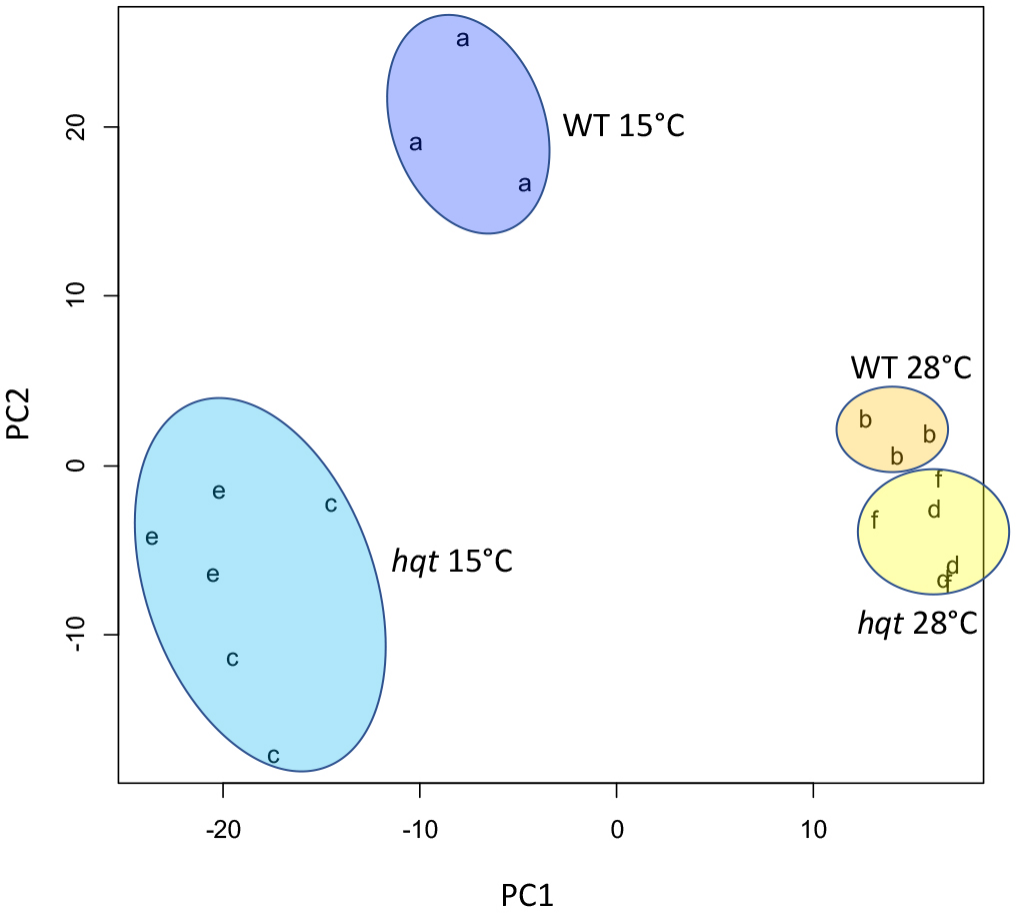
Untargeted metabolomic analyses of WT and *hqt* tomato plants grown at normal and low temperatures. Principal Component Analysis plot of LC/MS metabolomics of *hqt#3* (c, d)*, hqt#12* (e, f) and WT (a, b) tomato leaves at 28°C (b, d, f) and 15°C (a, c, e).

During cold acclimation tomato plants respond with several transcriptional and metabolic adjustments which include modulation of phenylpropanoid metabolism (Barrero-Gil et al., 2016). As these metabolic adjustments may involve both flavonoids and hydroxycinnamic acids, including CGA, we evaluated how *hqt* mutants respond metabolically to low temperature. With this aim we grew WT and *hqt* plants in growth chambers at 28°C and 15°C measuring any changes by metabolic profiling. We first undertook an untargeted analysis followed by PCA statistical analysis (**Figure 5**). PC1, the axis accounting for the greatest variability in the full dataset, could distinguish between cold and warm treatments as cold moves points to the left. PC2, included the second largest source of variation, and appeared to distinguish between the effect of cold on WT and on the two *hqt* mutants. At warm temperature (28°C) WT and *hqt* plants were similar but could be grouped in two different clusters. Mutant *hqt* lines appeared similar (to each other) under both conditions (28°C and 15°).

This analysis indicated that cold treatment caused a conspicuous change in the metabolite composition of leaves. PCA analysis also showed that low temperatures amplified the differences between WT and *hqt* lines, suggesting that an inability to make CGA could induce deep modifications in metabolite synthesis.

**Figure 6:**
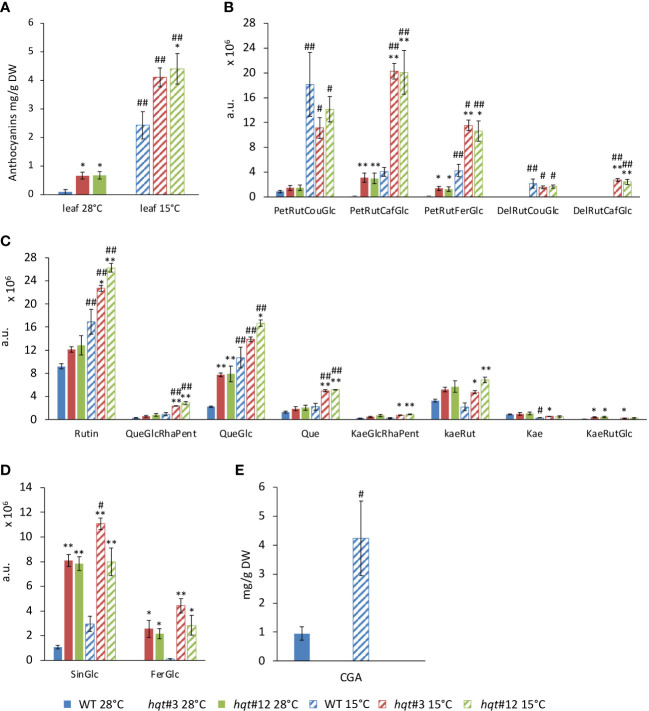
Targeted phenylpropanoid profiling of WT and *hqt* tomato plants grown at normal and low temperatures. Levels of total **(A)** and specific **(B)** anthocyanins, flavonols **(C)**, sinapoyl-glucose and feruloyl-glucose **(D)** and CGA **(E)** in leaves at 28°C and after 11 days at 15°C. The data show means ± SE (n = 3). Statistical significance was assessed by one-way ANOVA analysis followed by Dunnet’s tests for *hqt* lines vs WT, *P < 0.05, **P < 0.01 and by Bonferroni’s test for 15°C vs 28°C, ^#^ *P* < 0.05, ^##^ *P <*0.01. For CGA only 15°C vs 28°C comparisons have been made by Student’s t-test, ^#^ *P* < 0.05, ^##^ *P <*0.01. PetRutCouGlc, petunidin-rutinoside-coumaroyl-glucoside; PetRutCafGlc, petunidin-rutinoside-caffeoyl-glucoside; PetRutFerGlc, petunidin-rutinoside-feruloyl-glucoside; DelRutCouGlc, delphinidin-rutinoside-coumaroyl-glucoside; DelRutCafGlc, delphinidin-rutinoside-caffeoyl-glucoside; CGA, chlorogenic acid, 5-caffeoyl quinic acid; SinGlc, sinapoyl-glucose; FerGlc, feruloyl-glucose; Rut, rutin; QueGlcRhaPent, quercetin-glucose; QueGlc, quercetin-glucose; Que, quercetin; KaeGlcRhaPent, kaempferol-glucosyl-rhamnosyl-pentoside; KaeRut, kaempferol-rutinoside; Kae, kaempferol; KaeRutGlc, kaempferol-rutinoside-glucoside.

To explore better the metabolic change, we undertook a targeted analysis of selected flavonoids and hydroxycinnamic derivatives by LC/MS (**Figure 6** and **Table S5**), as well as total anthocyanin quantification (**Figure 6A**). First, we looked at CGA which was induced 4-fold by cold stress in WT plants (**Figure 6E**), whereas in *hqt* plants, it was completely absent at both 28°C and 15°C.

WT plants grown at 28°C accumulated anthocyanins poorly, whereas at 15°C their biosynthesis increased resulting in a 30-fold induction (**Figure 6A**). Notably, *hqt* plants showed ~8-fold and 1.75-fold higher levels of anthocyanins at 28°C and 15°C, respectively (**Figure 6A**). These data show that inactivation of the *HQT* gene results in a change in anthocyanin metabolism at both temperatures. LC/MS analysis confirmed that at 28°C WT plants produce tiny amounts of anthocyanins with PetRutCouGlc as the main compound (the other two petunidins were detected at negligible levels and the delphinidins were absent) (**Figure 6B**). After cold treatment, all petunidins were induced and PetRutCouGlc was the main compound; among delphinidins, only DelRutCouGlc was produced in the WT. These data indicate that anthocyanin acylation mainly occurs with coumaroyl-CoA as the acyl donor. At 28°C in *hqt* plants slightly more PetRutCouGlc accumulated than in WT but this could not explain the difference in the total anthocyanin amounts between WT and *hqt* mutants. Significantly higher levels of PetRutFerGlc and PetRutCaffGlc were found in *hqt* plants. Delphinidins remained undetectable. At 15°C PetRutFerGlc and PetRutCaffGlc accumulation was much higher in *hqt* mutants than in WT, but the levels of PetRutCouGlc were not significantly different in *hqt* plants compared to WT. Similarly, DelRutCouGlc levels did not change but DelRutCaffGlc was higher in *hqt* plants compared to WT plants. These data confirmed the increased accumulation of the total anthocyanins in *hqt* plants compared to WT plants at both temperatures and showed that loss of HQT activity impacts the acylation of anthocyanins, such that use of the caffeoyl group increases at the expense of other acyl-groups (especially the coumaroyl group).

Among flavonols, we looked at quercetin- and kaempferol-derived compounds (**Figure 6C**). The levels of rutin, the most abundant flavonol, at 28°C were slightly higher in *hqt* plants. The difference became stronger and statistically significant at 15°C. A similar trend was observed for QueGlcRhaPent, Que, KaeGlcRhaPent, KaeRut, Kae and KaeRutGlc. The difference was statistically significant only for QueGlc in both conditions, 28°C and 15°C.

We also found sinapoyl-glucose and feruloyl-glucose accumulated much more in *hqt* plants than in WT at 28°C as well as at 15°C (**Figure 6D**). Overall, these results indicated that in the absence of HQT activity and of CQAs, phenylpropanoid metabolism undergoes substantial reorganization especially under stress conditions.

### 3.5 Effect of CGA depletion on gene expression in leaves at normal and low temperature

**Figure 7:**
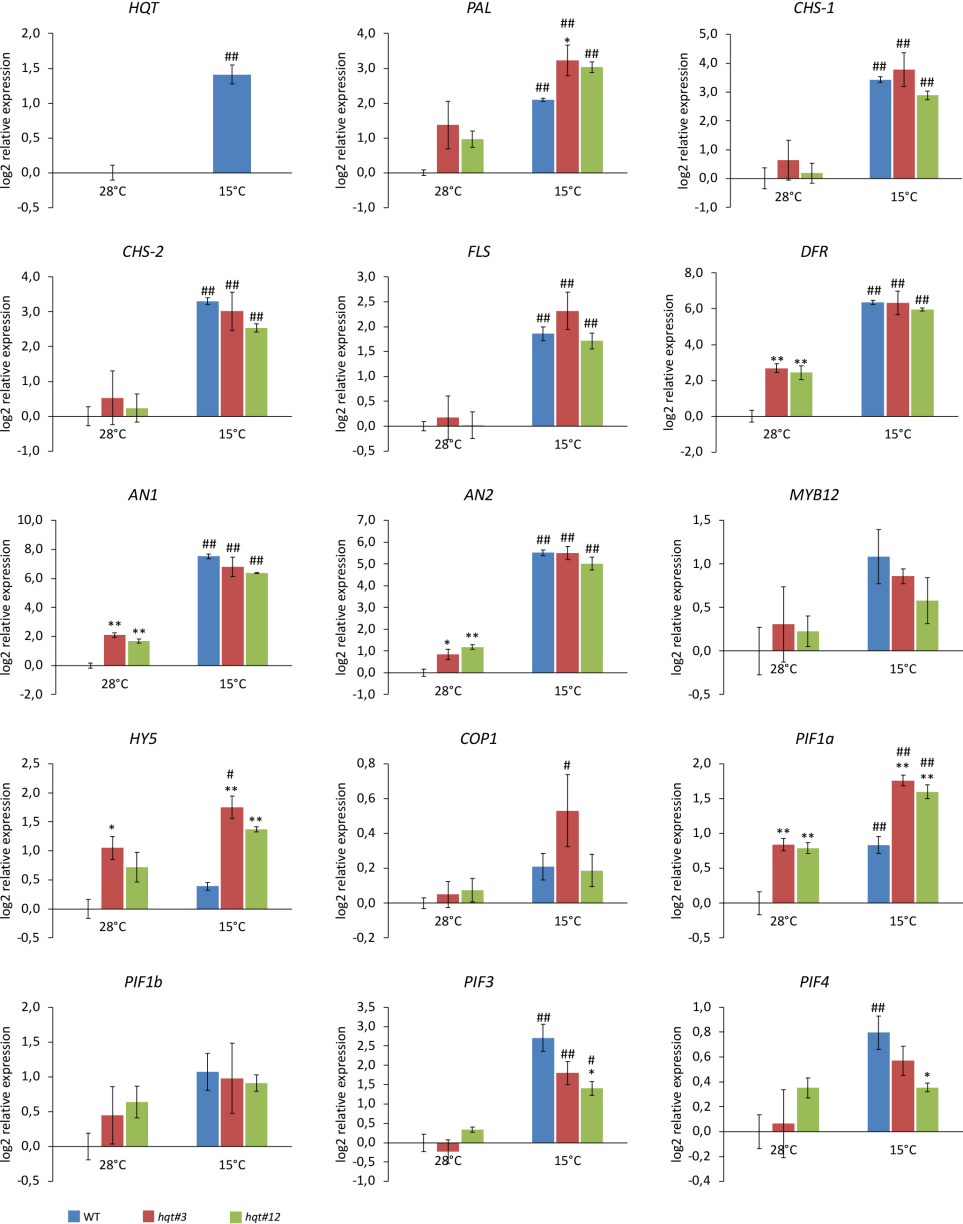
Expression analysis of genes encoding enzymes of the phenylpropanoid pathway and transcriptional regulators. WT and *hqt* tomato plants were grown at 28°C for 3 weeks and then subjected to cold stress for 11 days. Relative mRNA expression levels were referred to the untreated control. Data are means of relative quantification (Log2) of three biological replicates normalized to *SlEF1a* ± SE. Statistical significance was assessed by one-way ANOVA analysis followed by Dunnet’s tests for *hqt* lines vs WT, *P < 0.05, **P < 0.01 and by Bonferroni’s test for 15°C vs 28°C. ^#^ *P* < 0.05, ^##^ *P <*0.01. For *HQT* only 15°C vs 28°C comparison has been made by Student’s t-test, ^#^ *P* < 0.05, ^##^ *P <*0.01. For this experiment we used homozygous plants for *hqt#3A* allele and segregant plants from biallelic plant for *hqt#12A/B* alleles.

As anthocyanin production is induced by stress, we asked if the increased accumulation of anthocyanins in *hqt* plants was due to a rerouting of flux (from chlorogenic acid biosynthesis to anthocyanin biosynthesis) or due to the specific stress conditions which induced anthocyanin biosynthetic genes. Therefore, we analysed the expression of selected genes encoding enzymes of the phenylpropanoid pathway (*HQT*, Phenylalanine Ammonia Lyase (*PAL*), Chalcone Synthase-1 (*CHS-1*), *CHS-2*, Flavonol synthase (*FLS*) and Dihydroflavonol 4-Reductase (*DFR*)) as well as gene regulators (*AN1, AN2, MYB12, HY5, COP1, PIF1a, PIF1b, PIF3* and *PIF4*) involved in the regulation of phenylpropanoid pathway and responses to environmental signals (**Figure 7**).

At 28°C, we found that the expression of the *DFR* gene, which catalyses the conversion of dihydroflavonols to leucoanthocyanidins, was up-regulated in *hqt* plants (**Figure 7**). No other gene encoding an enzyme showed significant changes in expression in *hqt* plants; only *PAL* was, on average, upregulated in both *hqt* lines, (although without statistical significance). Interestingly, two transcription factors involved in the regulation of anthocyanin biosynthesis, *AN1* (bHLH) and *AN2* (MYB), were upregulated in *hqt* mutants. This was consistent with the higher accumulation of anthocyanins in *hqt* mutants and showed that the accumulation of anthocyanins in *hqt* mutants was not dependent (at least not completely) on rerouting of metabolic flux, but rather on the transcriptional activation of anthocyanin pathway by AN1 and AN2 possibly because of the *hqt* plants being stressed, even at 28°C. To explore this further, we analysed the expression of transcriptional regulators involved in stress and environment perception responses. Among them, only HY5 and PIF1a showed significant higher expression levels in *hqt* plants at 28°C (**Figure 7**).

In WT and *hqt* plants all genes encoding enzymes of the phenylpropanoid pathway and the transcription factors *AN1* and *AN2* showed increased transcript levels after 11 days of cold treatment. This result was consistent with the observed induction of accumulation of anthocyanin and other phenolic compounds.

In addition, some PIF genes (*PIF1a*, *PIF3* and *PIF4*) were significantly upregulated after cold treatment, although *HY5* and *COP1* transcript levels were only slightly higher at 15°C compared to 28°C. Overall these data suggested that the regulatory module HY5-COP1-PIFs is involved in cold adaptation in tomato.

At 15°C, *DFR*, *AN1* and *AN2* did not show any substantial differences in expression between WT and *hqt* mutant plants. This would suggest that the higher levels of anthocyanins in *hqt* mutants result primarily from a rerouting of metabolic flux rather than an upregulation of anthocyanin biosynthetic enzymes. However, *HY5* and *PIF1a* were upregulated in *hqt* mutants compared to WT. Interestingly, *PIF3* and *PIF4* were more weakly induced at 15°C in *hqt* plants than WT. All together these data indicate that *hqt* mutant plants could have impaired perception of the environment compared to WT. Overall, these data suggest that at lower temperatures *hqt* plants undergo transcriptional deregulation of stress/environment sensors and some key enzymes of the phenylpropanoid pathway to enhance the production of polyphenols in leaves.

### 3.6 Caffeoylquinic acids accumulate in the upper epidermis of leaves as a screen against UV-B light

The high concentration of CGA in leaves, especially under HL conditions in WT plants, is consistent with the protective role of phenolic compounds against harmful UV light and the scavenging of ROS formed as a consequence of excess light energy or UV-caused damage (Sharma et al., 2019). Typically, phenolics exert their protective functions because they accumulate in the upper epidermis and the upper layers of the mesophyll of leaves (Weissenböck et al., 1986; Hutzler et al., 1998; Hunt et al., 2021). Phenolic compounds can be found in several leaf cell types such as trichomes, epidermis, palisade and spongy mesophyll cells (Agati et al., 2013) and have been reported in several cell compartments: cell walls (Hutzler et al., 1998), vacuoles (Hutzler et al., 1998), nucleus (Hutzler et al., 1998; Polster et al., 2006) and chloroplasts (Saunders and McClure, 1976; Agati et al., 2007) possibly reflecting the wide spectrum of action of this heterogenous group of molecules in photoprotection of leaves. Typically, hydroxycinnamic acids (HCAs) accumulate in the vacuoles of epidermal and mesophyll cells (Agati et al., 2007; Agati et al., 2013). The accumulation of HCAs in cells exposed to light is consistent with a protective role by inhibiting the penetration of UV-B rays to the internal cells.

We investigated whether CGA accumulates differentially on upper and lower epidermis by comparing *hqt* plants to WT plants and evaluating whether the lack of CGA and the rearrangements of metabolism in *hqt* leaves have differential physiological impact in leaves following UV-B treatment.

Several classes of phenolic compounds show autofluorescence when irradiated with UV or blue light. To study the histolocalization of CQAs in tomato leaves, we used microspectrometric imaging by confocal laser scanning microscopy, and *hqt* mutant leaves to control for background signal.

**Figure 8:**
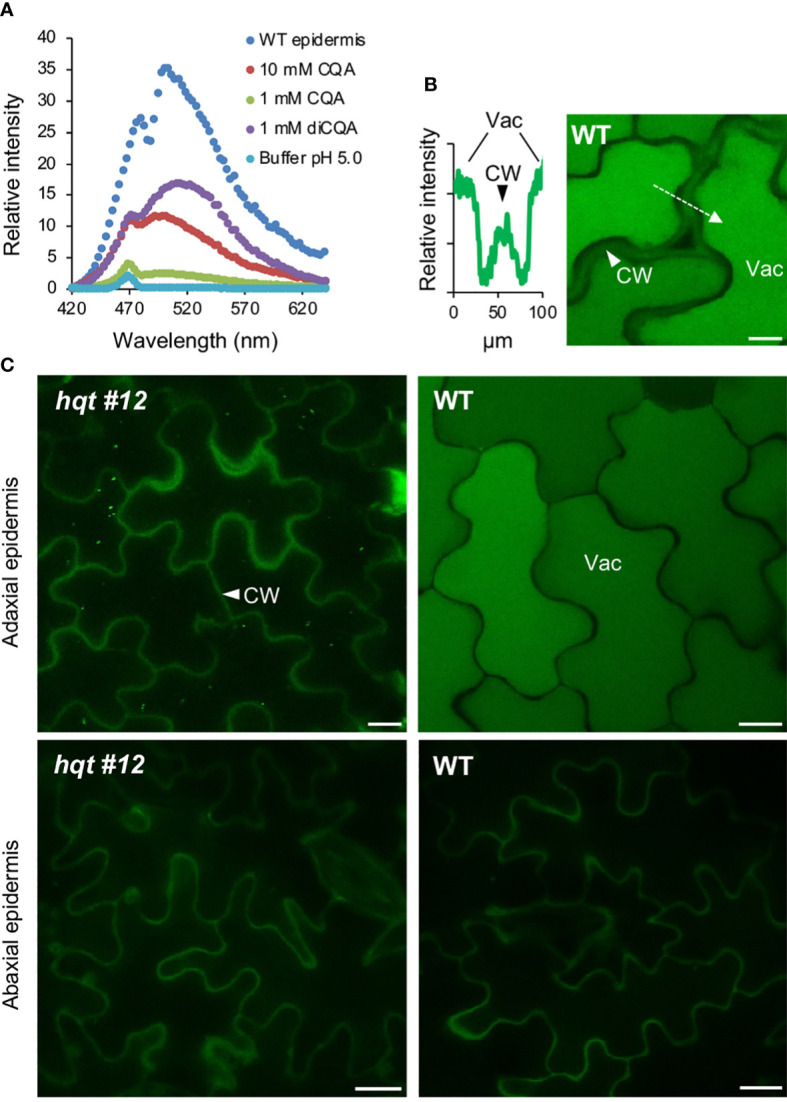
CQA/diCQA autofluorescence in leaves. **(A)** Emission spectra of CQA and diCQA standards at different concentrations in McIlvaine’s citrate-phosphate buffer at pH 5, after excitation at 405 nm. Cells in the upper epidermis of wildtype leaves of Moneymaker show the same spectrum as the standards. **(B)** Autofluorescence in cells of the adaxial and abaxial epidermis of Moneymaker and *hqt* mutant leaves after excitation at 405 nm. Lateral optical sections in the centre of pavement cells are shown. Fluorescence of cell walls was detected in all samples, whereas CQA/diCQA fluorescence was restricted to vacuoles in cells of the adaxial epidermis of wildtype leaves. **(C)** Relative fluorescence intensity (left panel) along the axis marked in the image, shown in the right panel. The optical section was taken in the apical part of the cells, showing autofluorescence in both, cell wall and vacuole. Scale bars represent 10 µm. CW, cell wall; Vac, vacuole.

As a result of excitation by UV light, the adaxial epidermal cells of WT leaves showed a strong signal in the vacuole (**Figures 8B, C**), with fluorescence in the emission band for CQA and diCQA standards (**Figure 8A**). The *hqt #12* leaf did not show any relevant signal in the upper epidermal cell vacuoles. Similar analysis of the abaxial epidermis showed no signal for either WT nor *hqt #12* mutant.

These results showed that CQAs accumulate in the vacuole of the adaxial epidermal layer cells, but not in the abaxial ones. This suggested that the accumulation of CQA on the light-exposed side of WT leaves could reflect its role in photoprotection by acting as a UV screen.

**Figure 9:**
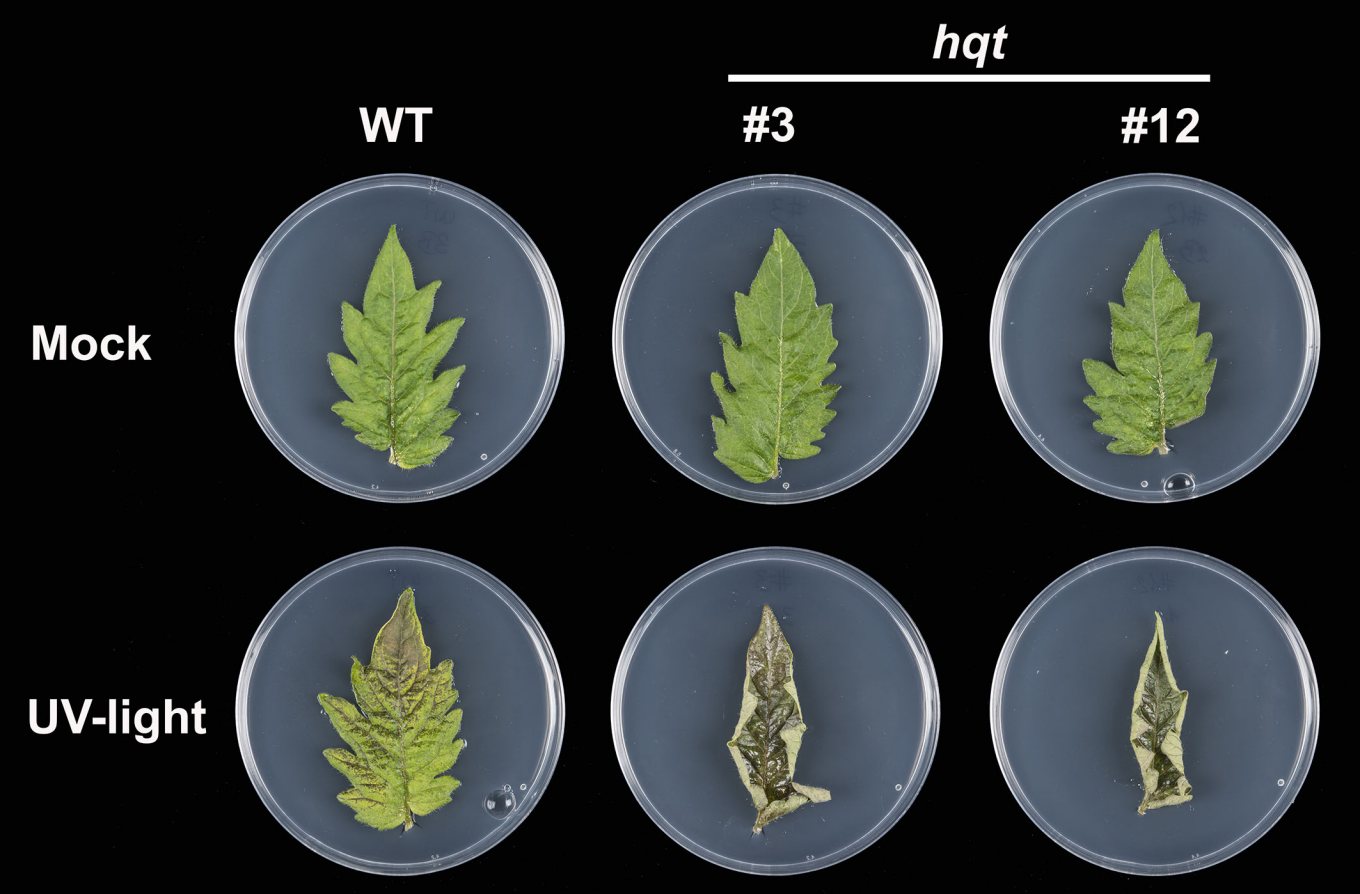
The effect of *hqt* mutation on UV-B resistance in tomato leaves. Primary leaflets were exposed to UV-B light (3.2 mW/cm^2^) for 30 minutes or kept in normal conditions (mock experiment). *hqt* mutants showed much higher sensitivity after UV-B exposure. No difference was observed in the mock experiment. Five replicates of WT and two independent mutants were used in this study.

We then assessed whether *hqt* mutants showed differential sensitivity to UV-B light (**Figure 9**). After a 30-minute exposure to UV-B light and 4 days recovery, WT leaflets showed a degree of damage with brown necrotic areas yet, overall, the leaflets were still green and vital. In contrast, the surface of *hqt* leaflets showed extreme injury apparently due to the death of the cells exposed directly to UV-B light. Moreover, an upward curling of *hqt* leaves occurred during the recovery period. The mock experiment (leaflets treated in the same way except for UV-B treatment) did not affect the appearance of WT or *hqt* leaflets.

These results demonstrated that the CQAs produced by HQT play an essential role in protecting tomato leaves against acute UV-B irradiation and the rerouting of CGA precursors towards the other phenylpropanoids in *hqt* mutants is not sufficient to compensate for the absence of CQAs.

## 4 Discussion

We generated tomato plants mutated in the *HQT* gene and undertook a characterization of the accumulation of CGA in WT and mutated plants, assessing metabolic and physiological effects. To the best of our knowledge, we show for the first time that the change in phenylpropanoid metabolism, as response to the absence of CGA, depends not only on the reallocation of hydroxycinnamates to other conjugates, but under certain environmental conditions, on changes in gene expression possibly caused by the changed stress state of *hqt* plants.

### 4.1 HQT-mediated CQA biosynthesis is the only relevant pathway producing CGA in tomato

The wide distribution and the high concentration of CGA in several organs of tomato highlight the importance of understanding the pathway for biosynthesis and its regulation. In plants three different pathways have been proposed to produce chlorogenic acid (**Figure 1**); in tomato and potato it has been demonstrated that the HQT-dependent way is predominant and that other pathways, if active, have relatively minor roles (Niggeweg et al., 2004; Payyavula et al., 2015). However, these conclusions were based on *HQT* silencing which leads to a strong reduction in CGA accumulation (over 90%) but not its elimination. To overcome the limitations of gene silencing and to study CGA biosynthesis more deeply in tomato, we generated tomato plants edited in the *HQT* gene (*hqt*). Some of the mutations introduced caused a complete loss of function of HQT and using these lines we were able to evaluate the contribution of HQT to CGA biosynthesis. We found that in two independent knock-out lines CGA levels were null or almost null establishing that the HQT pathway alone contributes significantly to CGA accumulation. Only in *hqt* leaves exposed to high light we were able to detect small amounts of CGA, estimated, maximally, as 1% of WT levels in the *hqt* mutant lines. These results indicated that, in tomato, another enzyme can synthesize CGA at very low efficiency. It is unlikely that the HCGQT-mediated way is active in tomato, as to date, no homolog encoding a hydroxycinnamoyl d-glucose: quinate hydroxycinnamoyl transferase (HCGQT) has been identified in the tomato genome. Considering the extremely low levels of CGA in *hqt* plants, it is likely that, in these plants, the residual production of CGA is HCT-dependent.

HQT and HCT are acyltransferase enzymes belonging to the same clade of the BADH protein family (D'Auria, 2006). HQT has substantially higher affinity for quinic acid than for shikimic acid as acyl acceptors (Niggeweg et al., 2004) and HCT prefers shikimic acid over quinic acid (Hoffmann et al., 2003). However, *in vitro*, they show a degree of acyl acceptor promiscuity. The HCT enzyme from coffee can catalyze the transesterification reaction between caffeoyl-CoA and quinic acid to give 5-CQA *in vitro* (Lallemand et al., 2012). Overall, we conclude that, in tomato, the HQT pathway is responsible for production of almost all CGA and that the direct contribution by HCT is negligible.

### 4.2 Role of CGAs in photosynthetically and non-photosynthetically active organs

Previously it has been shown that a reduction in CGA negatively affects the tolerance of tomato leaves to UV-B in terms of damage to the photosynthetic machinery (Clé et al., 2008). Here, thanks to confocal-based microspectrometric imaging, we were able to show that in tomato leaves CQAs accumulate only in the upper epidermis, highlighting that they provide a molecular shield on the light-exposed side of the leaf to block, at least in part, the UV-B rays and to mitigate their negative impact on plant cells. These results demonstrated unequivocally that CGA-free leaflets suffer much greater injury than WT leaves after UV-B exposure with upward curling of the leaf edges. This phenomenon has been also observed in UV sensitive Arabidopsis mutants, and might be due to inhibition of division or expansion of upper epidermis cells more than the lower epidermis cells (Li et al., 1993; Landry et al., 1995). In *hqt* plants the upper epidermal cells were strongly damaged, confirming that CGA in these cells makes an important contribution to mitigating the deleterious effects of UV-B rays and that this contribution can not be compensated by remodelling of the phenylpropanoid pathway in *hqt* mutants.

We also showed that CGA accumulates in photosynthetically active organs like stems and leaves and its accumulation is higher when leaves are exposed to high light indicating that CGA biosynthesis is regulated by light intensity. This is consistent with the evidence that under high light conditions, the total amount of phenolics increases and is accompanied by induction of several genes of the phenylpropanoid pathway (Linatoc et al., 2018; Pérez-López et al., 2018; Sutulienė et al., 2022). Photosynthesis is a vital process for the plant which requires protection from harmful UV light and from toxic molecules, like ROS, usually produced by plant cells during normal photosynthesis. Consequently, plants have developed several mechanisms to minimize UV penetration and to contain the damage caused by ROS. CGA absorbs UV-B light and is a powerful ROS scavenger, so its relatively high concentration in these organs is compatible with its roles in the control of the oxidative homeostasis by preventing the damage that ROS might cause to membranes, proteins and DNA and mitigating the penetration of interior cell layers by UV-B rays. Tomato fruits are photosynthetically active during fruit and seed development until the breaker stage when chloroplasts start to differentiate into chromoplasts and the fruit shifts from partially photosynthetic to heterotrophic metabolism (Carrari and Fernie, 2006; Quinet et al., 2019). We show that CGA levels gradually decrease during fruit development and ripening and this decrease is slower in the skin than the flesh. This is consistent with substantial metabolic rearrangements that occur during fruit development and ripening (Carrari and Fernie, 2006; Li et al., 2020; Zhu et al., 2022) and suggests that CGA accumulates when the photosynthetic apparatus is active, mainly in the outer layers of fruit to make a protective screen. In addition, CGA also accumulates in non-photosynthesizing organs like flowers and roots where it may contribute to maintaining oxidative homeostasis. In fact, tomato flowers require regulation of redox homeostasis as they produce and scavenge the ROS in a similar way to leaves (Rogers and Munné-Bosch, 2016). Therefore, the high concentrations of CGA in flowers might serve to control ROS levels especially under stress conditions and to mitigate the detrimental effects of UV-B light. In roots, in addition to ROS scavenging, CGA might be involved in heavy metal chelation since its content increases after exposure to toxic metals (Kısa et al., 2016; Soviguidi et al., 2022).

### 4.3 Metabolic rearrangements in *hqt* leaves are due to both redirection of metabolic flux and changes of gene expression

Previous work in tomato and potato has shown that phenylpropanoid metabolism undergoes remodeling in response to a reduction of HQT expression and accompanying changes in CGA accumulation, and that these changes depend on the environmental conditions and organ type (Clé et al., 2008; Payyavula et al., 2015). Consequently, a strong reduction or complete loss of CGA biosynthesis should lead to drastic changes at the metabolic and physiological levels. To test this hypothesis, we performed metabolic analysis on leaves from WT and *hqt* plants grown under different conditions: in the greenhouse and in growth chambers at warm and cold temperatures. Under these experimental conditions, we observed that hydroxycinnamoyl-glucoses accumulated more in *hqt* plants than WT. Sinapoyl-glucose, coumaroyl-glucose and feruloyl-glucose were more accumulated in greenhouse-grown plants and sinapoyl-glucose and feruloyl-glucose in growth chamber-grown plants (at both warm and cold conditions). We also observed that some flavonols accumulated more in *hqt* plants than WT, possibly due to a rerouting of flux from the hydroxycinnamic pathway to the flavonoid pathway, in agreement with the observations of Clé et al. (2008) and Payyavula et al. (2015). In addition, *hqt* plants grown in growth chambers produced more anthocyanins than WT. It is likely that the failure to use the caffeoyl-CoA precursor to produce CQAs in *hqt* plants redirects flux towards synthesis of both hydroxycinnamic acids and flavonoids. A possible explanation of the new metabolic status after HQT inactivation is that caffeoyl-CoA can be used by other enzymes, being converted in *p*-coumaroyl-CoA, feruloyl-CoA and sinapoyl-CoA. These, in turn, can be used for acylation reactions which explain the accumulation of the hydroxycinnamoyl-glucoses and the differential anthocyanin acylation in *hqt* plants.

Tomato plants usually use *p*-coumaroyl groups to acylate anthocyanins, as shown by the ratio between PetRutCouGlc and PetRutFerGlc and between PetRutCouGlc and PetRutCaffGlc in WT plants. Interestingly, in the *hqt* lines, we observed higher feruloylation and caffeoylation at the expense of *p*-coumaroylation. This might be due to feruloyl-CoA and caffeoyl-CoA being at higher concentrations than *p*-coumaroyl-CoA in *hqt* mutant lines.

We did not find any anthocyanin sinapoylation in either WT or *hqt* mutants, even though we detected relatively high amounts of sinapoyl-glucose (which showed, indirectly, that sinapoyl-CoA was produced); this was consistent with the observation that the arabidopsis and tomato anthocyanin acyl transferases belonging to BAHD family do not efficiently utilise sinapoyl-CoA (Luo et al., 2007; Tohge et al., 2015). Hydroxycinnamoyl-glucoses are usually stored in the vacuole and could be used for glycosylation and acylation of secondary metabolites by acyl-glucose dependent acyltransferase (Sasaki et al., 2014). Their increased accumulation in *hqt* plants has been observed in our growth conditions; however, whereas in greenhouse-grown plants we found high levels of coumaroyl-, feruloyl- and sinapoyl-glucose, in plants grown in growth chambers (under warm and cold temperatures) we found differences only for feruloyl- and sinapoyl-glucose, whereas no difference was detected for coumaroyl-glucose. This could be due to the fact that growth-chamber raised plants produce increased levels of anthocyanins, the biosynthesis of which depletes the pool of *p*-coumaroyl-CoA making it less available for coumaroyl-glucose biosynthesis.

The increased production of anthocyanins in *hqt* leaves, at least at 28°C, is likely due to the upregulation of the genes encoding DFR and the AN1 and AN2 transcription factors directly involved in anthocyanin biosynthesis. In addition, we found that the transcription factor HY5, which integrates several environmental signals, is also upregulated in *hqt* plants at 28°C. In Arabidopsis it has been shown that HY5 is involved in the control of ROS homeostasis in response to light, cold or nitrogen stresses (Catalá et al., 2011; Chen et al., 2013; Chai et al., 2015; Bellegarde et al., 2019). In tomato SlHY5 regulates anthocyanin production in CRY1a-dependent way directly binding *CHS-1*, *CHS-2* and *DFR* promoters (Liu et al., 2018). In addition, *AN1*, *AN2* and *ANT1* are upregulated in tomato plants overexpressing *SlHY5* and dowregulated in *SlHY5-RNAi* lines (Liu et al., 2018). More recently, Qiu et al. (2019) showed that SlHY5 positively regulates anthocyanin and flavonol biosynthesis in tomato hypocotyl and cotyledons of the Indigo Rose variety of tomato controlling the expression of structural genes of the pathway, including *PAL*, *CHS*, *CHI*, *F3H*, *F3’5’H*, *DFR*, *ANS*, *F3’H* and *FLS*. It can therefore be hypothesised that, due to the absence of the antioxidant CGA in the leaves, a higher oxidative state exists in *hqt* mutants than in WT, which could result in increased levels of HY5, which in turn could lead to an increase in anthocyanin biosynthesis.

Interestingly, at low temperature, while we observed increased levels of various phenolic compounds, anthocyanins, flavonols and hydroxycinnamic acid derivatives in *hqt* plants, no significant alterations in gene expression were observed for genes involved in these pathways compared to WT. This is probably due to the fact that many genes, including *DFR*, *AN1* and *AN2*, are strongly induced by cold stress, which obscures any differences in the expression of these genes in *hqt* compared to WT. One exception is the *PAL* gene whose expression showed a slight upregulation in *hqt* plants. It is well known that PAL controls overall amounts of phenolics by determining flux into the pathway (Bate et al., 1994; Howles et al., 1996). This suggests that the increased accumulation of phenolics in *hqt* plants occurs through the regulation of the PAL gene.

## 5 Conclusions

We carried out a characterization of tomato plants edited in the *HQT* gene resulting in knock-out alleles that caused the almost complete absence of CGA. This allowed us to clarify the role of HQT in the biosynthesis of the most abundant phenolic compounds in *Solanaceae* plants and to explore metabolic and physiological effects in tomato plants lacking CGA.

CGA accumulated in all organs analyzed, supporting the notion that it has a wide spectrum of action in tomato plants with its main involvement in the control of the oxidative homeostasis and in the protection against UV-B irradiation and pathogens. Plants lacking CGA showed very serious damage after UV exposure, suggesting that this molecule plays a pivotal role in protection against UV-B irradiation which can not be compensated by the overproduction of other compounds. However, all together metabolic and expression data indicate clearly that the absence of CGA in *hqt* plants can lead to a reorganization of phenylpropanoid metabolism involving both redirection of flux to other branches and remodeling of expression of important regulators which integrate internal and external clues to regulate metabolism and growth.

In the near future, it would be interesting to verify whether the overall metabolic and expression changes observed could give *hqt* plants a differential ability to tolerate low temperatures or other abiotic stresses when grown under UV-free light conditions, such as in the greenhouse or in soil-free systems.

## Data availability statement

The original contributions presented in the study are included in the article/**Supplementary Material**. Further inquiries can be directed to the corresponding author.

## Author contributions

CM conceived and supervised the research. LH undertook metabolite analysis. IA performed confocal-based microspectrometric imaging analysis. MP performed cold experiment. GM supervised the cold experiment and gene expression analysis. JL built the construct for genome editing. TL undertook tomato transformation. WH supervised tomato transformation. FD’O performed plant genotyping, UV experiments, sample processing, gene expression analysis, drafted the manuscript and prepared the figures with all authors contributing to and approving the final version. All authors contributed to the article and approved the submitted version.
