# Supplementary material for: Exploring the metabolic and physiological roles of HQT in S. lycopersicum by gene editing
Source: Front Plant Sci. 2023 Mar 31;14:1124959. doi: 10.3389/fpls.2023.1124959 (PMC10102458; doi:10.3389/fpls.2023.1124959)
Supplement: Supplementary file 1 [file Table_1.docx]

Supplementary Material

# Supplementary Tables – S1-S6

**Table S1**. Primers used for cloning

| **Primer name** | **Primer sequence** |
| --- | --- |
| sgHQT_A_FW | TGTGGTCTCAATTGAGAAAGAGCTAAAGAGATAGTTTTAGAGCTAGAAATAGCAAG |
| sgHQT_B_FW | TGTGGTCTCAATTGAAGTTAATTGTAATGGTGAGTTTTAGAGCTAGAAATAGCAAG |
| sgRNA_REV | TGTGGTCTCAAGCGTAATGCCAACTTTGTAC |

**Table S2**. Primers used for HQT and T-DNA genotyping

| **Primer name** | **Primer sequence** | **Allele/gene genotyped** |
| --- | --- | --- |
| SlHQT_-368_FW | TGATTCGAAATCAATTTAAAGAACAT | *hqt#12A, hqt#12B, hqt#3A* |
| SlHQT_389_REV | TAACATATAGGTAAATAAACACTAAG |  |
| SlHQT_-3386_FW | CCGGGTTATAGATTTTGGACGG | *hqt#3B* |
| qPCR_SlHQT_REV | TGATGAAGTGGATGGATGAGAG |  |
| SlHCT_FW | AGGTGAAAAACTCAACGATGGT | *HCT* |
| SlHCT_349_REV | GTCGCCGGAGCTCCAACGTCGG |  |
| Cas9_31_FW | TATCGGCACAAACAGCGTC | *Cas9* |
| Cas9_590_REV | CGAAAAGCTGATTGTAAGTCTG |  |

**Table S3**. Primers used for expression analysis by qPCR

| **Gene** | **Primer name** | **Primer sequence** | **Reference** |
| --- | --- | --- | --- |
| HQT Solyc07g005760 | qPCR_SlHQT_FW | GTGTTTTGTTTGTTGAGGCTGA | Luo et al., 2008 |
|  | qPCR_SlHQT_REV | TGATGAAGTGGATGGATGAGAG | Luo et al., 2008 |
| PAL | SlPAL_FW | AACCTATCTCGTGGCTCTTT | This work |
|  | SlPAL_REV | TCTTTTTCGCTGAATCTTGC | This work |
| CHS1 Solyc09g091510 | SlCHS1_FW | TGGTCACCGTGGAGGAGTATC | This work |
|  | SlCHS1_REV | GATCGTAGCTGGACCCTCTGC | This work |
| CHS2 Solyc05g053550 | SlCHS2_FW | CGGGCTACTAGGCAAGTTTTAAG | This work |
|  | SlCHS2_REV | CCTGTGGTACTAAGCCCTTCTTT | This work |
| FLS | SlFLS_FW | GAGCATGAAGTTGGGCCAAT | Bovy et al.,2002 |
|  | SlFLS_REV | TGGTGGGTTGGCCTCATTAA | Bovy et al.,2002 |
| DFR Solyc02g085020 | SlDFR_FW | TCCGAAGACGACAACGGTTT | Kiferle et al., 2015 |
|  | SlDFR_REV | TGACAAGCCAAGAGCCGATAA | Kiferle et al., 2015 |
| AN1 Solyc09g065100 | SlAN1_FW | CCTCTCTTGGACGGTGTTGT | Kiferle et al., 2015 |
|  | SlAN1_REV | GCTTGTTGTGGCTCATTGAA | Kiferle et al., 2015 |
| AN2 Solyc10g086250 | SlAN2_FW | TTCCAGGAAGGACAGCAAAC | Kiferle et al., 2015 |
|  | SlAN2_REV | AACGAGGACGAGAATGAGGA | Kiferle et al., 2015 |
| MYB12 Solyc01g079620 | SlMYB12_FW | GAGAAGGCTCTTGGAGGTCG | This work |
|  | SlMYB12_REV | GTCTACAACTCTTTCCGCATCTC | This work |
| HY5 Solyc08g061130 | SlHY5_FW | GCAAGCGACGAGTTCTAT | Wang et al., 2018 |
|  | SlHY5_REV | ATCTCCGGCACTCTTCTG | Wang et al., 2018 |
| COP1 Solyc12g005950 | SlCOP1_FW | TGGGACAGTGACAGAATGGG | Wang et al., 2019 |
|  | SlCOP1_REV | TTGGGATAGAGTTGACTGGTAG | Wang et al., 2019 |
| PIF1a Solyc09g063010 | SlPIF1a_FW | GCTCCGCCCATAAATCA | Rosado et al., 2016 |
|  | SlPIF1a_REV | GCTCCGCCCATAAATCA | Rosado et al., 2016 |
| PIF1b Solyc06g008030 | SlPIF1b_FW | TAGTATGGCAAAATGGTGGAG | Rosado et al., 2016 |
|  | SlPIF1b_REV | CGGCGTCACAACTCGGTG | Rosado et al., 2016 |
| PIF3 Solyc01g102300 | SlPIF3_FW | AAGGCTTCCCAATAATGC | Rosado et al., 2016 |
|  | SlPIF3_REV | CCATCAGACCAAACTTCCC | Rosado et al., 2016 |
| PIF4 Solyc07g043580 | SlPIF4_FW | GGCTTAGGTTCACATACAG | Rosado et al., 2016 |
|  | SlPIF4_REV | TGATGGTGTCGTTGTCTC | Rosado et al., 2016 |
| EF1a X14449 | SlEF1a_FW | GCTGCTGTAACAAGATGGATGC | Kiferle et al., 2015 |
|  | SlEF1a_REV | GGGGATTTTGTCAGGGTTGTAA | Kiferle et al., 2015 |

**Table S4.** Annotation of MS fragmentation patterns of selected compounds identified after untargeted metabolomics (plant grown in greenhouse)

| **Compound** | **Retention time min** | **Molecular ion (m/z)** | **MS/MS fragments (m/z)** |
| --- | --- | --- | --- |
| Sinapoyl-glucose | 5.47 | 409.11 [M+Na]^(+)^ | 247.06, 185.04 |
|  | 5.59 | 409.11 [M+Na]^(+)^ | 247.06, 185.04 |
| Coumaroyl-glucose | 4.011 | 349.09 [M+Na]^(+)^ | not detected |
|  |  | 344.13 [M+NH_4_]^(+)^ | 147.04, 165.05 |
|  |  | 325.09 [M-H]^(-)^ | 163.04 |
| Feruloyl-glucose | 5.018 | 379.10 [M+Na]^(+)^ | 185.04 |
|  |  | 355.10 [M-H]^(-)^ | 193.05 |
| Rutin | 7.061 | 633.14 [M+Na]^(+)^ | 331.10, 487.08 |
|  |  | 611.16 [M+H]^(+)^ | 303.05, 465.10 |
|  |  | 609.15 [M-H]^(-)^ | 301.04 |
| Chlorogenic acid | 4.54 | 377.08 [M+Na]^(+)^ | not detected |
|  |  | 355.10 [M+H]^(+)^ | 163.04 |
|  |  | 353.09 [M-H]^(-)^ | 191.06 |
| Unidentified, possibly terpenes | 9.885 | 393.31 [M+H]^(+)^ | 375.30, 266.25, 249.22 |
|  |  | 415.29 [M+Na]^(+)^ | not detected |
| Quercetin-glucose (possibly fragment of larger precursor) | 7.175 | 463.09 [M-H]^(-)^ | 301.04 [M-H]^(-)^ |
| Possible flavonoid | 6.85 | 741.19 [M-H]^(-)^ | 609.14 [M-H]^(-)^ |

**Table S5.** Annotation of MS fragmentation patterns of selected phenylpropanoid compounds in normal and cold stress conditions

| **Compound** | **Retention time min** | **Molecular ion (m/z)** | **MS/MS fragments (m/z)** |
| --- | --- | --- | --- |
| PetRutCouGlc | 6.655 | 933.27 | 317.06, 479.12 |
| PetRutCafGlc | 5.876 | 949.26 | 317.06, 787.21, 479.12 |
| PetRutFerGlc | 6.848 | 963.28 | 317.06, 801.23, 479.12 |
| DelRutCouGlc | 5.876 | 919.26 | 303.04, 757.20, 465.10 |
| DelRutCafGlc | 5.241 | 935.25 | 773.19, 303.05, 465.10 |
| CGA | 3.829 | 377.08 [M+Na]^(+)^ | 215.05, 359.07 |
| SinGlc | 4.38 | 409.11 [M+Na]^(+)^ | 185.04, 247.06 |
| FerGlc | 4.084 | 379.10 [M+H]^(+)^ | 185.04 |
| Rut | 7.028 | 611.16 [M+H]^(+)^ | 303.05, 465.10 |
| QueGlcRhaPent | 6.538 | 743.20 [M+H]^(+)^ | 611.16, 465.10, 303.05 |
| QueGlc | 7.138 | 465.10 [M+H]^(+)^ | 303.05 |
| Que | 8.968 | 303.05 [M+H]^(+)^ | not detected |
| KaeGlcRhaPent | 7.27 | 727.21 [M+H]^(+)^ | 287.05, 449.11, 595.17 |
| KaeRut | 7.933 | 595.17 [M+H]^(+)^ | 287.06 |
| Kae | 9.67 | 287.05 [M+H]^(+)^ | not detected |
| KaeRutGlc | 5.211 | 757.22 [M+H]^(+)^ | 449.11, 287.05 |

**Table S6.** Chlorogenic acid quantification in WT and *hqt* tomato plants by MS

| **CGA quantification (mg/g DW)** | | | | | | | |
| --- | --- | --- | --- | --- | --- | --- | --- |
|  |  |  |  |  | | Fruit (Br) | |
|  | Flower | Root | Stem | Leaf LL | Leaf HL | Flesh | Skin |
| WT | 9,18±0,47 | 4,38±0,16 | 0,63±0,09 | 1,31±0,27 | 15,59±3,77 | 0,40±0,04 | 0,75±0,09 |
| hqt#3 | trace* | trace* | 0 | 0 | 0,031±0,005 | 0 | 0 |
| hqt#12 | trace* | trace* | 0 | 0 | 0,029±0,006 | 0 | 0 |

**unquantifiable amount detected by MS*

*Mean ± Standard Error, n=3*

*LL: Low Light*

*HL: High Light*

*Br: Breaker*

# Bibliography

Bovy A, de Vos R, Kemper M, Schijlen E, Almenar Pertejo M, Muir S, Collins G, Robinson S, Verhoeyen M, Hughes S, et al. 2002. High-Flavonol Tomatoes Resulting from the Heterologous Expression of the Maize Transcription Factor Genes LC and C1. The Plant Cell.14:2509-2526.

Kiferle C, Fantini E, Bassolino L, Povero G, Spelt C, Buti S, Giuliano G, Quattrocchio F, Koes R, Perata P, et al. 2015. Tomato R2R3-MYB Proteins SlANT1 and SlAN2: Same Protein Activity, Different Roles. PLOS ONE.10:e0136365.

Luo J, Butelli E, Hill L, Parr A, Niggeweg R, Bailey P, Weisshaar B, Martin C. 2008. AtMYB12 regulates caffeoyl quinic acid and flavonol synthesis in tomato: expression in fruit results in very high levels of both types of polyphenol. The Plant Journal.56:316-326.

Rosado D, Gramegna G, Cruz A, Lira BS, Freschi L, de Setta N, Rossi M. 2016. Phytochrome Interacting Factors (PIFs) in Solanum lycopersicum: Diversity, Evolutionary History and Expression Profiling during Different Developmental Processes. PLOS ONE.11:e0165929.

Wang F, Zhang L, Chen X, Wu X, Xiang X, Zhou J, Xia X, Shi K, Yu J, Foyer CH, et al. 2018. SlHY5 Integrates Temperature, Light, and Hormone Signaling to Balance Plant Growth and Cold Tolerance. Plant Physiology.179:749-760.
